# Pregnancy rates after slow-release insemination (SRI) and standard bolus intrauterine insemination (IUI) – A multicentre randomised, controlled trial

**DOI:** 10.1038/s41598-020-64164-4

**Published:** 2020-05-07

**Authors:** Julian Marschalek, Christian Egarter, Elisabeth Vytiska-Binsdorfer, Andreas Obruca, Jackie Campbell, Philip Harris, Maarten van Santen, Bernd Lesoine, Johannes Ott, Maximilian Franz

**Affiliations:** 1 Department of Obstetrics and Gynecology, Medical University of Vienna, Waehringer Guertel 18-20, 1090 Vienna, Austria; 2 Kinderwunschzentrum Goldenes Kreuz, Lazarettgasse 16, 1090 Vienna, Austria; 3 Faculty of Health and Society, University of Northampton, Northampton, NN2 7AL UK; 4 Department of Gynaecology, Wrightington Hospital, Wigan, Lancashire WN6 9EP UK; 5 Private Office and Spermbank, Kriegsstrasse 216, 76135 Karlsruhe, Germany; 6 A.R.T. Bogenhausen, Prinzregentenstraße 69, 81675 Munich, Germany

**Keywords:** Reproductive disorders, Clinical trials

## Abstract

This multicentre, randomised, controlled cross-over trial was designed to investigate the effect of intra-uterine slow-release insemination (SRI) on pregnancy rates in women with confirmed infertility or the need for semen donation who were eligible for standard bolus intra-uterine insemination (IUI). Data for a total of 182 women were analysed after randomisation to receive IUI (n = 96) or SRI (n = 86) first. The primary outcome was serological pregnancy defined by a positive beta human chorionic gonadotropin test, two weeks after insemination. Patients who did not conceive after the first cycle switched to the alternative technique for the second cycle: 44 women switched to IUI and 58 switched to SRI. In total, there were 284 treatment cycles (IUI: n = 140; SRI: n = 144). Pregnancy rates following SRI and IUI were 13.2% and 10.0%, respectively, which was not statistically significant (p = 0.202). A statistically significant difference in pregnancy rates for SRI versus IUI was detected in women aged under 35 years. In this subgroup, the pregnancy rate with SRI was 17% compared to 7% with IUI (relative risk 2.33; p = 0.032) across both cycles. These results support the hypothesis that the pregnancy rate might be improved with SRI compared to standard bolus IUI, especially in women aged under 35 years.

## Introduction

Globally, 10–15% of couples of reproductive age are affected by infertility^1^. Following diagnosis of infertility, and evaluation of its causes, couples can be provided with information about their likelihood of achieving a spontaneous pregnancy, and their chance of pregnancy after different treatment options. Intrauterine insemination (IUI) is often the first step in infertility treatment for couples with unexplained infertility, low-grade endometriosis, sexual function disorders and low-grade male subfertility^2^. Pregnancy rates following artificial insemination are low^3^ and depend on several factors, including: age; reason for sub-fertility or infertility; absence/presence and type of ovarian stimulation and timing of insemination^3,4^ as well as the number of inseminated motile sperm^5,6^.

A large retrospective cohort study, covering more than 15,000 IUI-cycles, reported a mean pregnancy rate of 5.6% per cycle, and cumulative ongoing pregnancy rates after the third, seventh and ninth cycles of 18%, 30% and 41%, respectively^7,8^. These data clearly show the importance of developing new strategies for improving pregnancy rates after IUI. Most studies that have investigated methods for improving pregnancy rates after IUI have focused on the choice of clomiphene citrate (CC; now being superseded by letrozole) or recombinant follicle stimulating hormone (rFSH), with or without the use of gonadotropin-releasing hormone (GnRH) antagonists to stimulate ovulation^9–13^. Some studies dealt with the question of immobilisation versus immediate mobilisation following the insemination procedure - as immediate mobilisation might cause leakage of spermatozoa out of the uterus - and provided conflicting results^14–16^. However, only a few studies have dealt with changes to the IUI technique itself, or have questioned the application method^5^. One alternative to IUI is intratubal insemination (ITI), also known as fallopian tube sperm perfusion (FSP). This technique differs from IUI in that a higher volume of prepared semen is used (4 ml compared with ≤0.5 ml) and is introduced directly into the fallopian tubes^17^. The hypothesis is that the presence of a higher sperm density in the fallopian tubes at the time of ovulation is more likely to result in pregnancy; however, available evidence suggests that there is no clear benefit for ITI/FSP over IUI^18–20^. Another modified IUI application technique is slow release insemination (SRI), which was first described in 1992^21^. The authors hypothesised that a persistent low concentration of spermatozoa might prolong the period of potential fertilisation and thereby mimic physiological sperm transportation from the cervix to the fallopian tube. We have recently published data from two pilot randomised, controlled cross-over studies that indicate a statistically significant advantage of SRI over conventional bolus IUI^22^. The present, larger, multicentre trial was performed to clarify the effect of intra-uterine SRI on pregnancy rates in women designated for standard bolus IUI.

## Materials and Methods

### Study design and patient population

This multicentre, randomised, controlled cross-over trial was conducted in women with infertility and/or the need for semen donation who were eligible for IUI in 11 fertility centres across Europe. As already stated in a prior pilot study^22^, the cross-over design was chosen because it has been shown to give results comparable to those from studies with a parallel design, and, thus, to be a valid approach for infertility trials^23,24^.

Oral and written informed consent was obtained from all participants. The study was approved by the main ethics committee of the Medical University of Vienna (EK 1227/2012) and by all other local ethics committees. It was conducted in accordance with the Declaration of Helsinki and was registered in the Current Controlled Trials Register (registration number NCT02315040, 19/10/2014).

Patients were recruited between October 2012 and June 2017. Women were included if they fulfilled all of the following criteria:^22^ (i) primary or secondary infertility, defined as a couple’s failure to conceive after 12 months of attempting conception; (ii) age 20–40 years; (iii) tubal patency as diagnosed by hysterosalpingography, hystero-contrast-sonography (hycosy) or dye test with a maximum time interval between tubal testing and the woman’s enrolment into the study of 12 months; (iv) since the total motile sperm count is a major factor which influences pregnancy changes after IUI^25^, a minimum of 10 million motile sperm cells/ millilitre (mio/ml) after preparation; (v) infertility due to anovulation and/or endometriosis and/or the need for semen donation and/or unexplained infertility. In this context, unexplained infertility is defined as the absence of a definable reason for a couple’s failure to conceive after 12 months of attempting conception despite a detailed evaluation of ovulation, tubal and uterine abnormalities and male infertility factors.

Patients with uterine abnormalities, such as a septate uterus, were excluded.

A computerised randomisation programme was used initially to assign women to either the standard bolus IUI treatment or the SRI method. Women who failed to conceive in this first course of treatment were then allocated to the alternative method for the second treatment.

The primary outcome parameter was serological pregnancy defined as a positive beta human chorionic gonadotropin (hCG) test (in urinary or blood samples) two weeks after insemination. Information on the patient’s age, gravidity, cause of infertility, reproductive and concomitant medications was collected along with information on her partner’s age, sperm motility, sperm count and percentage of normal/abnormal sperm according to the analysis on the day of the SRI or IUI procedure. Details of any adverse events (AEs) were also recorded.

### Follicle monitoring and ovarian stimulation

In unstimulated cycles transvaginal sonography for follicular monitoring was performed depending on the anticipated ovulation between day 10 and14 of the menstrual cycle and was continued until a follicle size over 18 mm was reached. 35–38 hours after endogenous LH-surge or ovulation induction with human chorionic gonadotropin (hCG) 5,000 or 10,000IU intramuscularly (Pregnyl®, Merck Serono; Brevactid®, Ferring Pharmaceuticals; Choragon®, Ferring Pharmaceuticals) or chorionic gonadotropin alpha 250 or 500 μg subcutaneously (Ovitrelle®, Merck Serono) the insemination (IUI/SRI) was performed.

Controlled ovarian stimulation was performed either with human menopausal gonadotropin (Menopur®, Ferring Pharmaceuticals) or Follitropin alpha/beta (Gonal F®, Merck Serono; Puregon®, Merck Sharp & Drohne) or combined Follitropin alpha/Lutropin alpha (Pergoveris®, Merck Serono) 75 IU subcutaneously starting from day 3–5 of the menstrual cycle or with clomiphene citrate 50 mg from day 5–9. Follicular monitoring was started on day 10 until a follicle size over 18 mm was recorded. 35–38 hours after ovulation induction with hCG 5,000 or 10,000IU intramuscularly (Pregnyl®, Merck Serono; Brevactid®, Ferring Pharmaceuticals; Choragon®, Ferring Pharmaceuticals) or chorionic gonadotropin alpha 250 or 500 μg subcutaneously (Ovitrelle®, Merck Serono) the insemination (IUI/SRI) was performed. Cycles were cancelled if more than 2 follicles over 16 mm were present. In stimulated cycles women received either vaginal progesterone 200 mg once daily or dydrogesterone 10 mg twice daily for 14 days.

### Sperm preparation method

The sperm preparation method was performed according to the World Health Organisation laboratory manual for the examination and processing of human semen (2010) either with density gradient preparation (n = 144/284) or swim up procedure (n = 140/284) from good quality samples using commercially available media (GM501 SpermAir - Gynemed GmbH & Co. KG, Lensahn, Germany; Gynemed Gradient 45%/90% - Gynemed GmbH & Co. KG, Lensahn, Germany; Origio sperm preparation medium – Origio, Måløv, Denmark).

Density gradient preparation: Application of each 1–1.5 ml of 45% and 90% gradient on the bottom of a conical test tube. Centrifugation of the test tube between 500–1,500 rpm, depending on sperm-counts, for 20–30 minutes (24–210xg). Aspiration of the pellet and resuspension with 2–5 ml of buffer. Centrifugation at 1,500–2,000 rpm for 10 minutes (210–380xg). Evaluation of sperm concentration and progressive motility.

Swim up procedure: Transfer of 1.5 ml of buffer into a test tube at a temperature of 25–36 °C. Injection of 0.5 ml of the sperm sample with a sterile 1 ml syringe in the bottom of the test tube. Incubation of the test tube for 15 to 30 minutes at a temperature of 35–36 °C.

Lift of the supernatant with motile sperm cells and transfer to a sterile test tube. Evaluation of sperm concentration and progressive motility.

### Standard bolus IUI technique

The standard bolus IUI is accomplished as described elsewhere^22^ with a polyethylene insemination catheter (5 French, 28 cm). Before performing the IUI, the catheter is joined to a 1 ml tuberculin syringe containing laboratory-prepared sperm. This is connected to the insemination catheter, which is inserted into the uterine cavity. After the injection of sperm and removal of the catheter, the patient is able to leave the clinic.

### SRI technique

**Figure 1:**
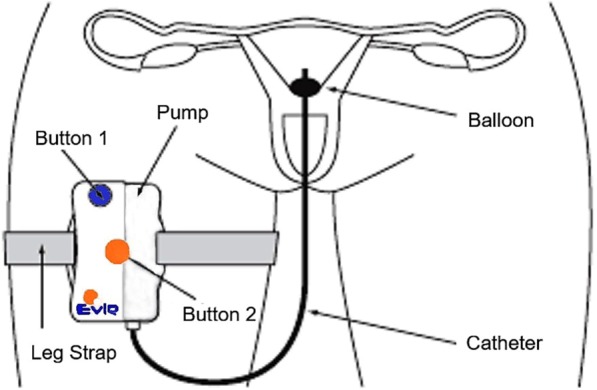
Schematic Illustration of the EVIE Slow Release Insemination Pump. (Figure by courtesy of Fertiligent Ltd.).

In this study, the EVIE device was used for women undergoing SRI. The device consists of a disposable EVIE syringe pump (Fertiligent, Ra’anana, Israel), a 3 ml sterile syringe (Becton Dickinson; Franklin Lakes, NJ) and a customised HSG catheter with inflatable anchor balloon at the tip (Catheter Research Inc; Indianapolis, IN). The disposable pump (Fig. 1) is a mechanical device in which a spring pushes the syringe plunger with a patented spring restriction mechanism; this allows it to keep running during the four-hour delivery period. Prepared sperm solution is held in a sterile, sperm-compatible syringe in the chamber of the EVIE device before being delivered through the catheter over four hours. The insertion of the catheter into the uterus is performed as for standard IUI. In contrast to the standard IUI procedure, however, after positioning the EVIE catheter into the uterine cavity, it is anchored in the uterus by using normal saline solution (1 ml) to fill the balloon positioned at the end of the catheter. The pump is strapped to the patient’s thigh and the device is activated by pressing button marked number 1. The patient is able to remain mobile (completely ambulatory) during the four-hour insemination procedure. There is no need for her to rest, and she may leave the clinic to go home. Once four hours has elapsed, button number 2 is pressed. This completes the procedure by flushing any remaining sperm in the syringe into the uterus. The patient removes the catheter herself by opening the white stopcock to empty the balloon before withdrawing the catheter. The entire device is then discarded.

### Sample size calculation

The *a priori* sample size for the study was calculated using a minimum clinically significant effect defined as a relative risk of 2.0 in favour of SRI and an estimated pregnancy rate for IUI for women aged <40 years of 14.3% (which was observed in an unpublished pilot data set). Using these estimates, a minimum sample size of 137 treatment cycles per group (treating each insemination as a separate case) was required for a superiority trial with a power of 90% and a significance level of 0.05.

### Statistical analysis

Data for a maximum of two cycles (one SRI and one IUI per woman) only were analysed. Data from patients who withdrew due to adverse effects or device failure, or who failed to comply with the protocol, were treated as missing. Because of the nature of the study design, each treatment (rather than each person) was considered as an independent case.

For numerical parameters, variables are summarised as either mean ± standard deviation (SD) or median with inter-quartile range (IQR) depending on the data distribution. For categorical parameters, data are presented as frequencies and percentages.

Chi-squared or Fishers’ exact tests were used to compare categorical variables between the two groups and independent-samples median tests were used to compare non-parametric numerical variables. One-tailed z-tests were used to test for superiority (i.e. relative risk [RR] of >1) of the pregnancy rate of SRI over IUI.

IBM SPSS 22.0 (SPSS Inc; Chicago, IL) was used for statistical analysis. P-values of less than 0.05 were considered statistically significant.

### Ethical approval

The study was approved by the main ethics committee of the Medical University of Vienna (EK 1227/2012) and by all other local ethics committees. All procedures performed in studies involving human participants were in accordance with the ethical standards of the institutional and/or national research committee and with the 1964 Helsinki declaration and its later amendments or comparable ethical standards. The study was registered in the Current Controlled Trials Register (registration number NCT02315040).

### Informed consent

Oral and written informed consent was obtained from all participants.

## Results

**Figure 2:**
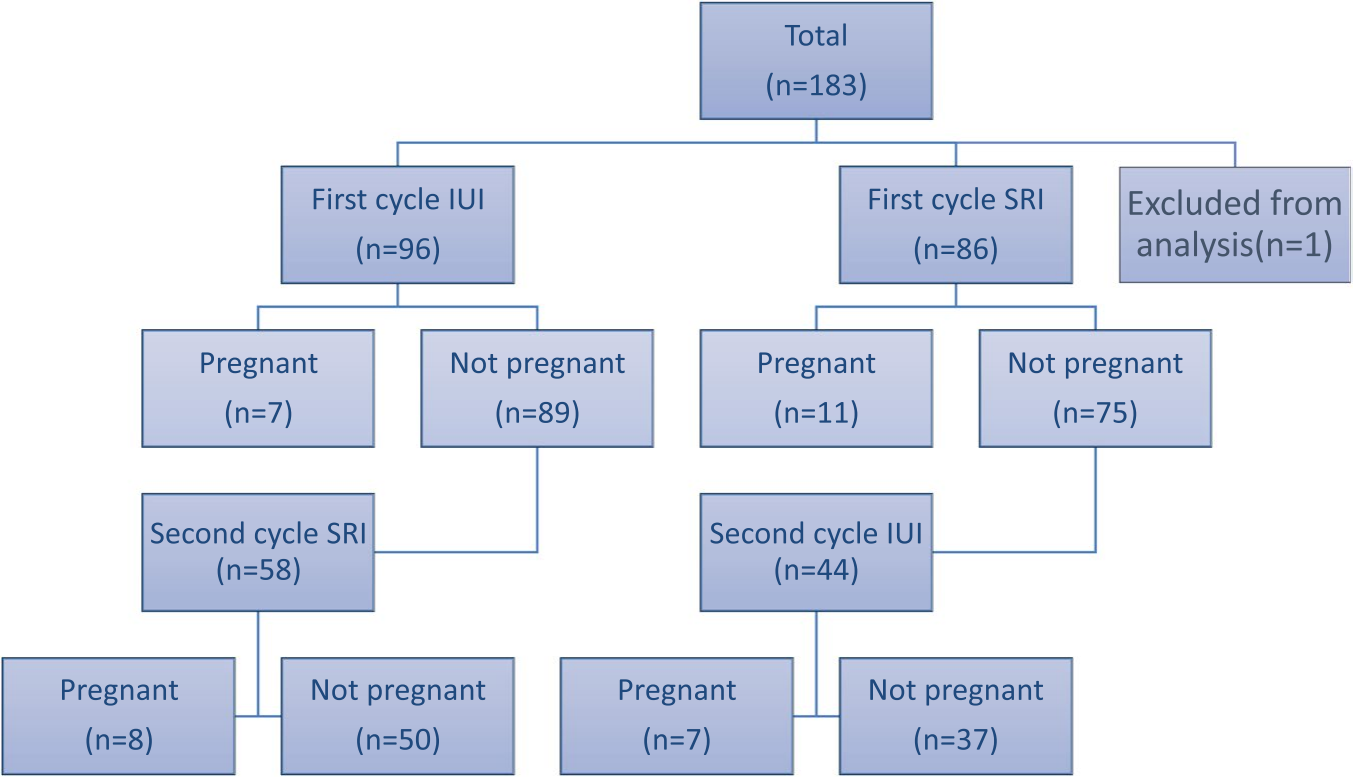
Flow chart showing the initial randomisation to IUI or SRI, pregnancy rates and numbers of patients crossing over to the alternate procedure. (IUI, intrauterine insemination; SRI, slow-release insemination).

A total of 183 women were randomised into the two treatment arms (Fig. 1): 96 (52.5%) women received standard IUI treatment first and 87 (47.5%) women received SRI treatment first. The EVIE device did not fully actuate in one patient assigned to the SRI group: she received a bolus at the end of the 4-hour administration period and her data were excluded from the analysis. Characteristics for analysed patients and their partners are shown in Table 1: there were no significant differences between the two groups. The median age of women participating in the study was 33 years (IQR 30–36 years). Donor sperm was used in 37 treatments for 23 women (13%). 102 women underwent a second treatment cycle (IUI: n = 44; SRI: n = 58; Fig. 2). In total, there were 284 treatment cycles (IUI: n = 140; SRI: n = 144).

**Table 1:** Baseline characteristics of all couples undergoing IUI and SRI procedures.

	IUI procedures (n = 140)	SRI procedures (n = 144)	p-value^b^
Female age, years*	33 (30-36)	33 (30-36)	0.840
Body Mass Index (kg/m^2^)*	22 (20-24)	22 (20-24)	0.899
Previous pregnancies			
0^#^	81 (58%)	91 (63%)	0.511
1^#^	47 (33%)	39 (27%)	
>1^#^	12 (9%)	13 (9%)	
Cause of infertility			
Endometriosis^#^	10 (7%)	11 (7%)	0.672
Anovulation^#^	22 (16%)	27 (19%)	
Unexplained infertility^#^	108 (77%)	105 (73%)	
Donor Insemination^#^	19 (14%)	18 (13%)	0.789
Reproductive Medication^‡^			
Clomiphene	48	49	0.963
Gonadotropins	89	90	0.852
Progesterone	33	33	0.896
Concomitant Medication			
Levothyroxine	9	8	0.757
Other^c^	4	6	0.549
Male age, years*	36 (33–40)	35.5 (32–39)	0.468
Semen analysis^a^			
Concentration (mio/ml)^*^	45 (17-77)	40 (15-77)	0.468
Motility (%)^*^	55 (45-75)	54 (45-73)	0.519
Normal morphology (%)^*^	19 (6-75)	12 (5-80)	0.394
Treatment cycle			
1^#^	96	86	0.120
2^#^	44	58	

IUI, intrauterine insemination; SRI, slow-release insemination.

Data are presented as *median (interquartile range) or ^#^n (%).

^‡^Reproductive medication – data presented as cumulative reproductive medication, multiple entries possible.

^a^Semen analysis – after preparation.

^b^Median test (numerical data); chi-squared/Fishers’ exact test (categorical data).

The observed overall serological pregnancy rate for SRI was 13.2%, compared to 10.0% for IUI (relative risk [RR] = 1.32); however, this difference was not statistically significant (95% confidence interval [CI] 0.69–2.53; p = 0.202).

### Effect of age on pregnancy rates

In total, 172 inseminations were performed in patients aged under 35 years (IUI: n = 83; SRI: n = 89). For all women aged under 35 years undergoing SRI, the observed RR for pregnancy was 2.33, with 16.9% of the SRI procedures resulting in pregnancy (n = 15) compared to 7.2% of the IUI procedures (n = 6). This difference was statistically significant (p = 0.032). There were no significant differences with respect to female and male characteristics for patients aged under 35 years in the IUI and SRI subgroups (Table 2). Total pregnancy rates following SRI and IUI are provided in Table 3.

**Table 2:** Baseline characteristics of couples undergoing IUI and SRI procedures where the woman was aged under 35 years.

	IUI procedures (n = 83)	SRI procedures (n = 89)	p-value^b^
Female age, years*	31 (28–33)	30 (28–33)	0.864
Body Mass Index (kg/m^2^)*	21 (20–24)	22 (20–24)	>0.999
Previous pregnancies			
0^#^	51 (62%)	62 (70%)	0.498
1^#^	26 (31%)	21 (23%)	
>1^#^	6 (7%)	6 (7%)	
Cause of infertility			
Endometriosis^#^	4 (5%)	4 (4%)	0.490
Anovulation^#^	16 (19%)	24 (27%)	
Unexplained infertility^#^	63 (76%)	61 (69%)	
Donor Insemination^#^	11 (13%)	9 (10%)	0.521
Reproductive Medication^‡^			
Clomiphene	26	29	0.860
Gonadotropins	54	59	0.865
Progesterone	22	24	0.946
Concomitant Medication			
Levothyroxine	4	6	0.590
Other^c^	2	3	>0.999
Male age, years*	34 (30–37)	34 (29–37)	0.759
Semen analysis^a^			
Concentration (mio/ml)^*^	45 (22–78)	40 (15–76)	0.398
Motility (%)^*^	54 (43–76)	52 (43–69)	0.291
Normal morphology (%)^*^	17 (7–70)	10 (5–72)	0.263
Treatment cycle			
1^#^	55	57	0.760
2^#^	28	32	

IUI, intrauterine insemination; SRI, slow-release insemination.

Data are presented as *median (interquartile range) or ^#^n (%).

^‡^Reproductive medication – data presented as cumulative reproductive medication, multiple entries possible.

^a^Semen analysis – after preparation.

^b^Median test (numerical data); chi-squared/Fishers’ exact test (categorical data).

**Table 3:** Pregnancy rates following slow release insemination and intrauterine insemination.

Insemination method	Pregnancy rate (%)
All women	11.6
SRI	13.2
IUI	10.0
Women <35 years	12.2
SRI	16.9*
IUI	7.2

IUI, intrauterine insemination; SRI, slow-release insemination.

*Statistically significant.

### Safety

There were no significant differences in the proportions of women reporting adverse events with the SRI procedure compared to the IUI procedure (7/144 vs. 1/140, p = 0.071) (Table 4). Two patients who experienced pain during catheter insertion, and one patient with syncope did not proceed with SRI treatment, but all other patients who reported adverse events did. No serious adverse events were recorded. With respect to pregnancy related complications there were 3 ectopic pregnancies reported following SRI and none following IUI. However, this difference was not statistically significant (p = 0.248) (Table 4).

**Table 4:** Adverse events.

Event	IUI procedure (n = 140)	SRI procedure (n = 144)
Post-interventional spotting	1	3
Pain during catheter insertion	0	2
Pain and cramps during procedure	0	1
Vasovagal syncope	0	1
Multiple pregnancies	0	0
Ectopic pregnancies	0	3

IUI, intrauterine insemination; SRI, slow-release insemination.

## Discussion

In this paper we report the results of the largest, randomised, controlled trial of a modified IUI technique, which was first described in 1992^21^.

In standard IUI, a bolus of highly-concentrated spermatozoa is delivered directly into the uterine cavity near to the fallopian tubes to increase the density of capacitated spermatozoa near the presumed oocyte, thereby optimising the chance for pregnancy^17^. However, a proportion of the spermatozoa are expelled through the fallopian tubes into the peritoneal fluid^26,27^ when a volume of 0.5 ml is used. This means that there is scope for improvement in this technique.

The concept underlying SRI is that a smaller number of spermatozoa continuously released into the uterus over an extended period of time will prolong the period of potential fertilisation, more closely mimicking physiological continuous sperm transportation into the fallopian tubes^22^. It could be hypothesised that this prolonged usage of the insemination balloon catheter in SRI also improves the transport of spermatozoa along the fallopian tubes by stimulating local prostaglandin production as a result of the pressure on the endocervix. However data about this hypothesis is very weak. Moreover, there is literature postulating a negative effect of inseminating too much sperm cells all at once^5,28^. These papers place this in the context of excessive reactive oxygen species (ROS) formation and multiple fertilization of the oocyte if too many sperm cells are present in the uterus or oviduct. Therefore slow release insemination with its duration of 4 hours and only inseminating few spermatozoa per minute could avoid this effect.

Muharib et al. were the first to report an improvement of pregnancy rates using this technique compared to the standard bolus technique^21^. A re-analysis of their data indicated that this difference failed to reach statistical significance (p = 0.057)^22^. A subsequent meta-analysis of data from the Muharib et al. study and two pilot studies demonstrated that SRI was statistically significantly more effective than IUI (RR 2.64; 95% CI 1.04–6.74; p = 0.02)^22^.

This randomised, controlled, multicentre trial demonstrated an overall numerically – but not statistically significant – higher pregnancy rate with the SRI procedure. In the subgroup of women aged under 35 years, pregnancy rates were significantly higher with the SRI procedure. Of note, women under 35 years of age are routinely referred for IUI, as falling pregnancy rates in older women might lead to earlier IVF treatment.

It is well known that pregnancy rates decrease with female age, whether or not artificial insemination is required^29–31^. In their much larger study in patients undergoing IUI, Schorsch et al. reported statistically significantly higher pregnancy rates for women below the age of 25 compared to women aged 35 years and over (p < 0.001), and they concluded that age was an important factor in achieving pregnancy after IUI^31^. The impact of male and female age on pregnancy rates after SRI needs to be analysed in further studies.

To exclude a bias on pregnancy rates by different stimulation methods within the treatment groups, we documented the method of stimulation (Tables 1 and 2). While generally there was no difference in the use of CC, rFSH or progesterone within the groups, an exceptionally high pregnancy rate in patients aged less than 35 years using CC and undergoing SRI was found (24,1%). Overall, however, no significant differences in the use of medications could be discovered in the treatment of women who became pregnant after SRI or IUI.

Values for absolute pregnancy rates with IUI differ widely in the literature; however, we could not find any other modification of the method or medication for IUI that had as strong an effect on pregnancy rates as SRI. This observation shows the potential benefit of SRI. IUI is a cost-effective method for increasing pregnancy rates in couples with unexplained infertility, which is why IUI is often chosen as first-line therapy in these patients^32^. One could speculate that an improvement in success rates with SRI might further reduce the number of referrals for more expensive assisted reproductive technology, and hence reduce the overall costs of infertility treatment; however, carefully designed clinical trials would be required to prove this.

We are aware that the cross-over design might be seen as a major limitation of this study. As already stated cross-over studies with repeated interventions on the same patient are not traditionally recommended for trials where a successful outcome will have a permanent serial effect (in this case, pregnancy) that results in the withdrawal of the patient from the second arm of the trial^22^. Pregnancy after the first treatment unbalances the research design and introduces a period effect. For this reason, some authors reject the utilisation of this study design in infertility trials^33,34^; however, others claim that it is an efficient and pragmatic design, particularly as only one cycle of each treatment is given to each woman^23,24^. To show this effect in our study, we evaluated the data from the first treatment course in patients aged under 35 years.

During the first cycle, 112 inseminations were performed in these patients; of these, 55 were randomised to the IUI procedure, and 57 to SRI. 5.5% and 14%, respectively, resulted in pregnancy (RR = 2.57). This difference was not statistically significant (95% CI for RR 0.72–9.20; p = 0.073); however, this can be explained by a lack of statistical power due to the small number of treatments (actual power = 46%). Cross-sectional studies have been shown to be a valid approach in infertility research:^23^ Takada *et al*. recently reported that the crossover design has the highest power and the smallest bias^24^. These authors recommended using a combination of a cross-over design and the Mantel-Haenszel method for two-period, two-treatment clinical trials with irreversible endpoints.

The mentioned considerations about sample size must also be included into the discussion about study limitations. The power calculation was done for the whole study population and not for women aged <35 years. Thus, the population size in the latter group is small and the analysis of this subgroup seems vulnerable to chance. This issue could have been solved by including women of younger age only which has not been the case.

The lack of information on previous infertility treatments and the use of donor sperm may additionally be seen as another limitation of the study. Moreover, two different types of sperm preparation methods may introduce bias. There were approximately equal numbers of IUI and SRI (19 IUI, 18 SRI) performed with donor sperm. This study, however, mainly included couples with unexplained infertility and not subfertile men, as sperm quality inclusion criteria were very strict. In this context we have to state that the sperm volume was not evaluated which we consider as minor study limitation.

The wide use of CC in the present study also must be discussed critically, as we are aware that this might be considered controversial. In accordance with the guidance from the Practice Group of the American Society for Reproductive Medicine, the relatively high rate of anovulation in both groups was addressed by administering CC^35^. The guidance notes that CC used in combination with IUI in cases of unexplained infertility seems to be beneficial. Up to three cycles is considered a common therapeutic regimen before progressing to more aggressive therapies. Last not least, despite the small sample size to achieve, the study period was quite long. Empirically, this was due to the fact that women did not accept IUI well and often opted for IVF instead. Moreover, the study was performed in 11 different centres around Europe which might have introduced some kind of unknown bias. However, since all centres performed IUI and SRI with similar frequency, we believe that this circumstance was of minor relevance. Interestingly, the only ectopic pregnancies reported in this study were following the SRI procedure. Althoug this difference was not statistically significant, the population size of this group is too small to draw any conclusions concerning safety regarding this point.

## Conclusion

In conclusion, these data lend support to the hypothesis that the pregnancy rate might be improved by using SRI rather than IUI, especially in women aged less than 35 years. Due to the above-mentioned study limitations, this trial should be seen as an additional pilot study which can also serve as a basis for future trials. Thus, additional, larger, clinical trials are required to fully prove the hypothesis of SRI’s superiority, especially if an economic benefit of SRI is also to be demonstrated, but also to evaluate potential adverse effects of this procedure with respect to the occurrence of ectopic pregnancies.
